# Numerosity comparison, cognitive strategies, and general cognitive functioning in older people

**DOI:** 10.3389/fpsyg.2024.1340146

**Published:** 2024-04-02

**Authors:** Jakub Słupczewski, Małgorzata Gut, Jacek Matulewski, Adam Tarnowski

**Affiliations:** ^1^Doctoral School of Social Sciences, Nicolaus Copernicus University, Toruń, Poland; ^2^Institute of Psychology, Faculty of Philosophy and Social Sciences, Nicolaus Copernicus University, Toruń, Poland; ^3^Department of Informatics, Faculty of Physics, Astronomy and Informatics, Nicolaus Copernicus University, Toruń, Poland

**Keywords:** numerical cognition, cognitive strategies, aging, cognitive functioning, memory, attention, visual–spatial abilities

## Abstract

**Introduction:**

Studies have shown age-related differences in numerical cognition, for example, in the level of numerosity comparison ability. Moreover, some studies point out individual differences in the cognitive strategies employed during the performance of numerosity comparison tasks and reveal that they are related to the aging process. One probable cause of these differences is the level of cognitive functioning. The aim of our study was to determine the relationships among numerosity comparison ability, the cognitive strategies utilized in the performance of numerosity comparison tasks and the general cognitive functioning in older people.

**Methods:**

Forty-seven elderly people participated in the study. The participants were examined using overall cognitive functioning scales and computerized numerosity comparison task.

**Results:**

The results showed many correlations between the participants’ level of cognitive functioning and the percent of correct responses (PCR) and response time (RT) during numerosity comparison, as well as with the cognitive strategies applied by the participants. Task correctness was positively related to the level of performance in the attention and executive function tasks. In contrast, the long-term memory resources index and visuospatial skills level were negatively correlated with RT regarding numerosity comparison task performance. The level of long-term memory resources was also positively associated with the frequency of use of more complex cognitive strategies. Series of regression analyses showed that both the level of general cognitive functioning and the cognitive strategies employed by participants in numerosity comparison can explain 9–21 percent of the variance in the obtained results.

**Discussion:**

In summary, these results showed significant relationships between the level of cognitive functioning and proficiency in numerosity comparison measured in older people. Moreover, it has been shown that cognitive resources level is related to the strategies utilized by older people, which indicates the potential application for cognitive strategy examinations in the development of new diagnostic tools.

## Introduction

The increasing age of the world population (Stewart et al., 2003) has raised an urgent need for research on cognitive function during the aging process. The need for research in this area is indicated by many publications, which indicate that getting older involves the loss of many cognitive functions, such as memory (Bläsi et al., 2009), attention (Quigley et al., 2010), and executive functions (Libon et al., 1994), to varying degrees. Moreover, the development of methods that will enable early and effective prediction and diagnosis of cognitive deficits that occur with age has become necessary. Early detection of cognitive decline allows for the administration of fast intervention, which can reduce the adverse effects of a disease (Pais et al., 2020). These methods are particularly important today because population-based studies suggest that between 50 and 80% of older people with normal cognitive test results report certain forms of perceived decline in cognitive functioning when they were asked during examination (Jessen et al., 2010; van Harten et al., 2018).

Numerical skills (including basic numerical competences, such as numerosity comparison) are an important aspect of everyday life, e.g., during shopping or paying bills. The concept of basic numerical abilities refers to primary abilities related to understanding and manipulating numbers and mathematical concepts. These abilities include understanding number relationships, comparing numbers and other basic mathematical operations and numerosity estimation (Geary, 2000). Moreover, these abilities are important for the development of several other more complex mathematical abilities, e.g., calculation, which is a part of our culture (Chen and Li, 2014). People constantly need to calculate, understand proportions and ratios, and remember much information related to numbers [telephone numbers or personal identification numbers (PINs)]. Some researchers show that reduced mathematical abilities lead to lower income and less financial security in everyday life (Butterworth et al., 2011). According to numerosity comparison, several studies have demonstrated age-related differences in the performance of such tasks (Cappelletti et al., 2014; Norris et al., 2015). A study described by Halberda et al. (2012), with more than 10.000 participants aged 11–85 showed that this ability begins to decline after age 30, which is probably attributed to a deficit in executive functions (Gandini et al., 2009). In another study, Roquet and Lemaire (2019) compared abilities in numerosity comparison among younger (aged between 18 and 26) and older people (aged between 65 and 93). The participants were shown a series of two dot sets and asked to select the largest set. The number of dots ranged from 12 to 48 in each group. Moreover, dot sets were displayed in four conditions that differed in the level of congruency. The authors observed congruency effects in both age groups (poorer performance in incongruent items relative to congruent items). Other results showed that older people were slower and less accurate overall with larger congruency effects than young adults. Research on the basic numerical skills of older people is important as this decrease can be an early predictor of dementia (Deloche et al., 1995) and Alzheimer’s disease (Mantovan et al., 1999). In healthy elderly people, we observe a decline in arithmetical functions (Zamarian et al., 2007). However, few studies focus on the diagnostic and therapeutic value (e.g., vis cognitive training) of numerical skills in a group of people at risk for cognitive deficits. The increasing importance of research on numerical skills in the aging process is apparent, among other things, regarding the new research methods that are being developed in this field, e.g., the Numerical Activities of Daily Living (NADL) (Semenza et al., 2014).

People use different cognitive strategies during numerical tasks, also in numerosity comparisons. In accordance with the definition proposed by Anderson (2005), cognitive strategy is understood as a controlled method of information processing that is used to achieve a specific cognitive purpose. Roquet and Lemaire (2019) distinguished nine strategies that differ in their complexity, such as strategies based on the distance between dots or the distance and size of the dots. Most of the strategies mentioned in the study focused on three visual aspects of the presented sets of objects: their size, the distance between them and the total area of the presented sets of elements. Older people are more likely to choose less effective strategies and use them less efficiently (Roquet and Lemaire, 2019). Gandini et al. (2008) demonstrated that older and younger people who choose the same strategies have different forms of implementation, which result in accuracy and the time of implementation of these strategies – younger adults were more accurate and faster (Gandini et al., 2008). The issue of whether systematic age-related differences in strategy selection during numerosity comparison tasks can be observed remains unclear. The suggested reason for age-related differences in strategy selection during numerosity comparison tasks is a decline in older people’ ability to quickly process information (Salthouse, 1996). Examining the strategic aspects of participants’ performance is a promising approach to understanding age-related differences and similarities in human cognition (Lemaire and Arnaud, 2008).

To the best of our knowledge and based on our familiarity with the related literature, no study has combined measurement of the numerosity comparison task with simultaneous measurement of cognitive strategies and cognitive functioning of participants. The need for further research in this area is also pointed out by the authors of the first known study of cognitive strategies among the elderly people (Roquet and Lemaire, 2019). The aim of our study was to determine the relationships among numerosity comparison ability, cognitive strategies employed in the performance of such tasks and the general cognitive functioning in older people. The examination of the present relationships was intended to determine whether the designed research paradigm can be useful in the diagnosis of cognitive deficits in aging. Given previous research indicating that higher cognitive functioning is related to more complex cognitive strategies during task execution, we predicted that participants with normal or higher cognitive resources should use cognitive strategies connected with two or more visual features more often than people who are experiencing decline in cognitive functioning. Given that cognitive methods measure many features of cognitive functioning, we also aimed to identify cognitive abilities that are connected to numerosity comparison ability. Due to the experimental nature of our study, we also aimed to verify the level of difficulty of the programmed task to assess numerosity comparison ability in a group of elderly people – in particular, the differences among the developed difficulty variants.

Based on the results of previous studies and our own research assumptions, we formulated the following research hypotheses:

*H1*: There are significant differences in the performance of the different variants of the task difficulty shown in terms of response time (RT) and percent of correct responses (PCR).

*H2*: Higher levels of cognitive functioning (more points on the MoCA and MMSE scales) are associated with faster and more accurate numerosity comparison.

*H3*: A lower level of cognitive functioning (less points on the MoCA and MMSE scales) correlates with the use of more simple cognitive strategies during numerosity comparison tasks.

## Materials and methods

### Participants

A group of 50 elderly people (aged 62–79) participated in the study. The participants belonged to local groups of older people, including listeners of the University of the Third Age. Three participants were excluded from the analyses because their score on the Mini-Mental State Examination scale (MMSE, Folstein et al., 1975) were lower than 26 points. All 47 participants who were included in the analysis had no vision and hearing problems and no relevant neurological or psychiatric disease (e.g., depression). The mean age of participants was 70.45 years (SD = 4.63). Among the participants, 41 were female and 6 were male. The participants varied in terms of education level (25 participants had higher education, 20 participants had secondary education and 2 participants had vocational education).

### Procedure

The tests were administered by an experienced researcher in the same order, starting with the MMSE (Folstein et al., 1975), followed by the Montreal Cognitive Assessment (MoCA, Nasreddine et al., 2005) and then a computerized task to evaluate numerosity comparison competence and cognitive strategies. All tasks were completed during one session, which lasted between 60 and 90 min. Before beginning the cognitive tests, each participant read information about the study and gave written and conscious consent to participate. The study was approved by the Local Ethical Committee of the Faculty of Philosophy and Social Sciences (Nicolaus Copernicus University in Torun) and conducted in accordance with the principles of the Declaration of Helsinki.

### Materials (only heading)

#### Mini-mental state examination

The mini-mental state examination (MMSE) (Folstein et al., 1975) is a pen-and-paper test of cognitive functioning. The total possible score is 30 points. The cut-off score between people with normal cognition and those with abnormal cognition is 26. The MMSE includes cognitive domain tests of orientation in time and space, attention, verbal memory, language abilities (e.g., naming) and visuospatial skills. It takes approximately 10–15 min to complete this test. The Polish version of this test was used in the study.

#### Montreal cognitive assessment

The Montreal cognitive assessment (MoCA) (Nasreddine et al., 2005) is a pen-and-paper test that is designed as a screening instrument for cognitive functions assessment. The total possible score is 30 points – a score of 26 or above is considered normal. The MoCA includes cognitive domain tests of attention, memory, language, executive functions, visuospatial ability, calculations, orientation in time and space and conceptual thinking. The test takes approximately 10 min to complete. The Polish version of this test was used in the study.

### Numerosity comparison task

**Figure 1:**
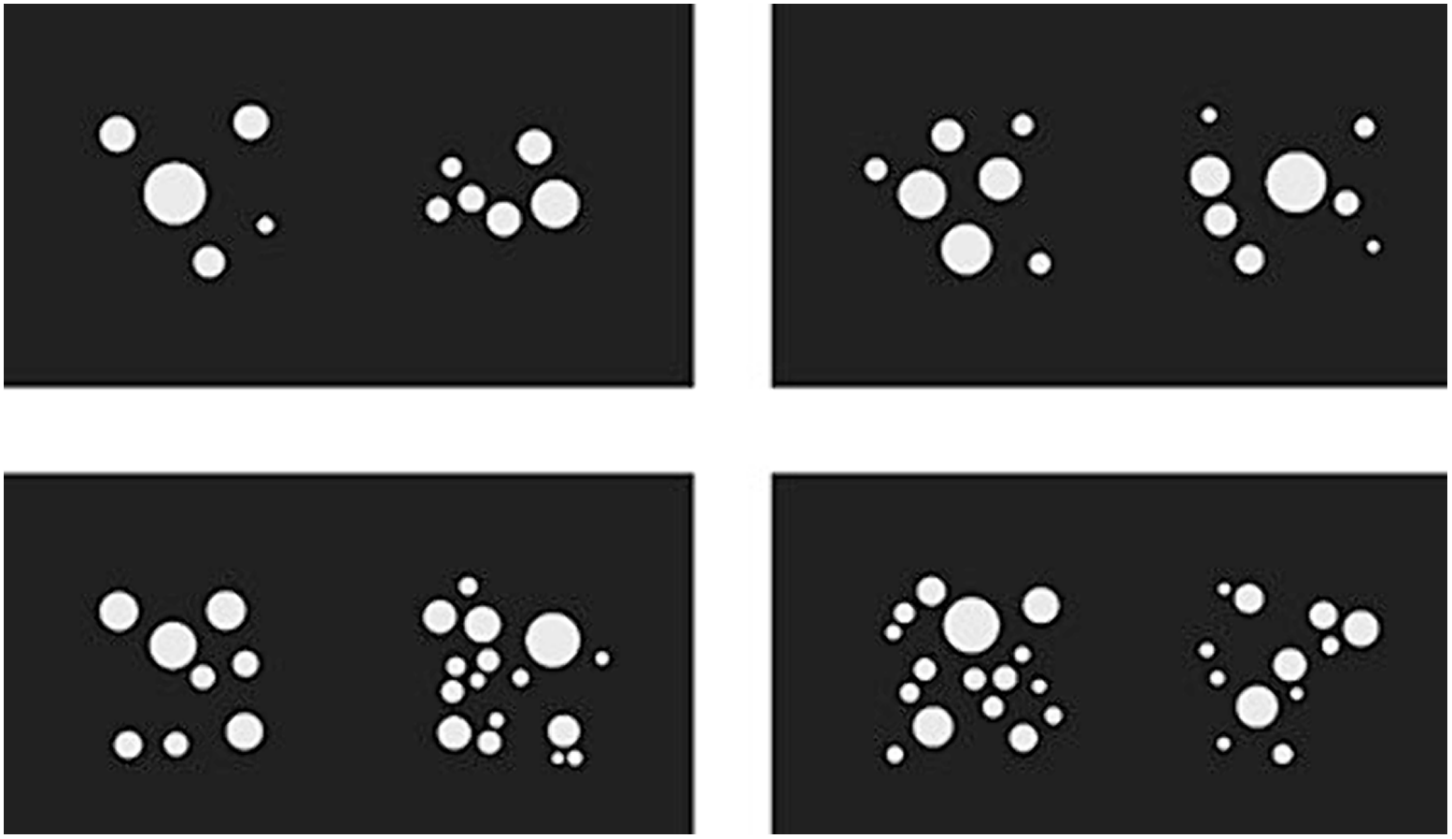
Examples of stimuli used to measure the numerosity comparison ability in the study paradigm (in different ratio variants).

**Figure 2:**
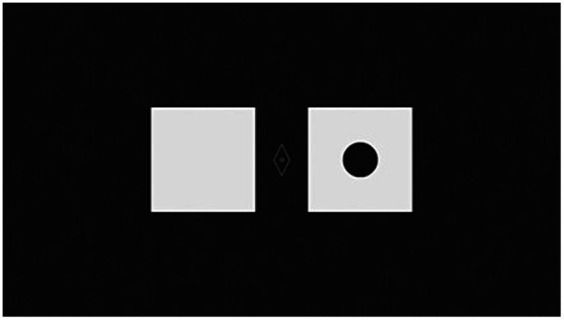
Example of stimuli used to measure simple RT in the study paradigm.

During the task, two sets of dots were presented on a computer screen, equidistant from the center of the screen. The participant’s task was to estimate without counting and then select as quickly as possible the side where, according to him, there were more elements. The number of dots in the sets ranged from 5 to 16. The number of sets in the selected task guaranteed the maintenance of numerous parameters at the correct and controlled level. The parameters included the average total area of dots, the area and perimeter of the convex hull around the dots, density (ratio of dots’ area per convex hull area), the dots’ center, and the averaged dot size. The differences in the assumed parameters were not larger than 2% (Gebuis and Reynvoet, 2011). The numerical task selected for the study enabled measurement of both RT and the PCR. Participants were informed that both parameters were equally important. Moreover, participants were asked to estimate the numerosity of each collection of dots, rather than exactly counting them (in situations where the RT was too long during the trial session, participants were again reminded not to count the set of dots presented). The instructions also included information about the need to describe the strategies used to select the larger of the sets of items. The exact content of the instructions is provided in the Supplementary materials. The participant had to press one of two buttons on a keyboard placed on the desk in front of him – “Z” (to select a set of dots on the left side of the screen) or “M” (to select a set of dots on the right side of the screen). Importantly, when responding with the keyboard, the buttons adjacent to the “Z” and “M” keys were also operative to avoid delays associated with imprecise keystrokes. The trials further differed in their difficulty, which was determined by the proportion of dots in the two sets that were presented. These proportions were 1:2, 3:4, 5:6, and 7:8 (from easiest to the most difficult) (see examples in Figure 1). In the main part of the procedure, the number of trials was 160 (40 trials for each level of difficulty). Before proceeding to the proper part of the test, the participant performed a trial part consisting of 20 samples. Moreover, at the beginning of the numerical task, the participants were asked to perform a short task to test simple RT [ms], which also consists of 20 samples (see examples in Figure 2). The participant’s task was to choose the square in which the black dot stimulus would appear as quickly as possible. This procedure was employed to familiarize the elderly people with how to answer using a computer keyboard. In this task, the “Z” and “M” keys were also used to give responses. There was no time limit for the presentation of sets of dots on the screen or when describing verbally the applied cognitive strategy. Between each trial, a fixation cross was presented in the center of the screen for the participant to view while verbally answering questions about cognitive strategy. Due to the large number of trials, the task was divided into five parts – the first trial with 20 stimulus pairs and the four blocks of trials with 40 stimulus pairs for comparison each. The larger set of elements was presented in equal numbers on the right and left side of the screen. The order of the tasks that were presented was random, but the tasks were presented in the same sequence for each participant.

### Cognitive strategies interview

While performing the numerosity comparison task, participants were asked to provide a verbal description of their choice of set of dots. While giving instructions, the participant was informed that after each of her/his responses (selection of a larger set of items), the researcher would ask the following question: “Based on which visual features did you select this set of dots?” To ensure that the participant understood the purpose of the task, the sample features - the size of the dots, the area of the set of dots, the distances between the dots and the shape of the whole - were given in the same order each time. Furthermore, as the researcher pointed out, the participant could make decisions based on other or more features than was mentioned. All responses were recorded on a recorder for later analysis. According to previous studies and the responses that were collected from the participants, 10 categories were listed according to which the answers were coded: 0 – lack of cognitive strategy (e.g., participant declares counting or is unable to name his strategy), 1 – distance between dots (e.g., “there were longer distances between dots”), 2 – size of dots (e.g., “there were more smaller dots”), 3 – total surface area of dots (e.g., “there were larger areas of white”), 4 – shape (e.g., “there were sets of dots that resembled squares”), 5 – size of dots and distance between them (e.g., “there were larger dots but with shorter distances between them”), 6 - size of dots and shape (e.g., “there were smaller dots that resembled comets”), 7 – size and total surface area of dots (e.g., “there were smaller dots but they covered a larger part of the computer screen”), 8 – total surface area and distance between dots (e.g., “there were larger breaks between dots and they covered a larger field”) and 9 – size of dots, distance between them and total surface area (e.g., “the dots were smaller, but there were larger distances between them so they covered a larger area”). The participants had an unlimited amount of time to present the cognitive strategies that they utilized.

### Apparatus and software

The stimuli were presented on a 23.8″ computer screen with a resolution of 1920 × 1,080 px and a 60 Hz refresh rate. The desktop computer used in the study was provided with an Intel(R) Core(TM) i3-10105T CPU processor and a 64-bit Windows 11 system. The stimulus presentation and the recording of participants’ responses were controlled, designed and implemented by Jacek Matulewski’s software, which was developed in the Microsoft Visual Studio 2017 Enterprise integrated development environment using C# language. The. NET Framework 4 Client Profile and Windows 7 or higher version was required.

### Data analysis

Analyses were performed with IBM SPSS (version 29.0). *p*-values below 0.05 were considered significant. Normality of the variables was measured with the Shapiro–Wilk test. The normal distribution of the variables was confirmed only for the following variables: overall score on the MoCA scale (*W* = 0.951, *p* = 0.053), accuracy of the 7:8 difficulty variant (*W* = 0.956, *p* = 0.079) and RT for the entire task (*W* = 0.955, *p* = 0.073) and in the 3:4 difficulty variant (*W* = 0.951, *p* = 0.051) and 5:6 difficulty variant (*W* = 0.956, *p* = 0.079). The results related to cognitive strategies represent the proportion calculated for each participant (given as a percentage) of the strategies, which been utilized relative to all the trials performed during the task. Descriptive statistics on the frequency of use of each cognitive strategy by the participants are included in Supplementary Table S2. The Student’s *t* and Mann–Whitney’s tests were conducted to analyze group differences among quantitative variables (e.g., RT and PCR in numerosity comparison). We use also the Students’s *t* and Wilcoxon’s tests to verify differences in the difficulty ratio variants. Pearson’s and Spearman’s correlation tests were conducted to analyze the relationships among variables (cognitive functioning level, numerosity comparison ability and cognitive strategies). We also use regression analysis methods to estimate the relationships between dependent variables (RT or PCR in numerosity comparison task) and independent variables (level of overall cognitive functioning or results as specified in cognitive domain tests, e.g., memory, attention or cognitive strategies). Those stimulus pairs whose numerosity comparison time exceeded the second standard deviation calculated for an individual results were excluded from the statistical analyses. Descriptive statistics of the MMSE, MoCA and numerosity comparison task scores reported in the study are included in the Supplementary Table S1.

## Results

### Differences between time and correctness of responses in different difficulty ratio variants

**Figure 3:**
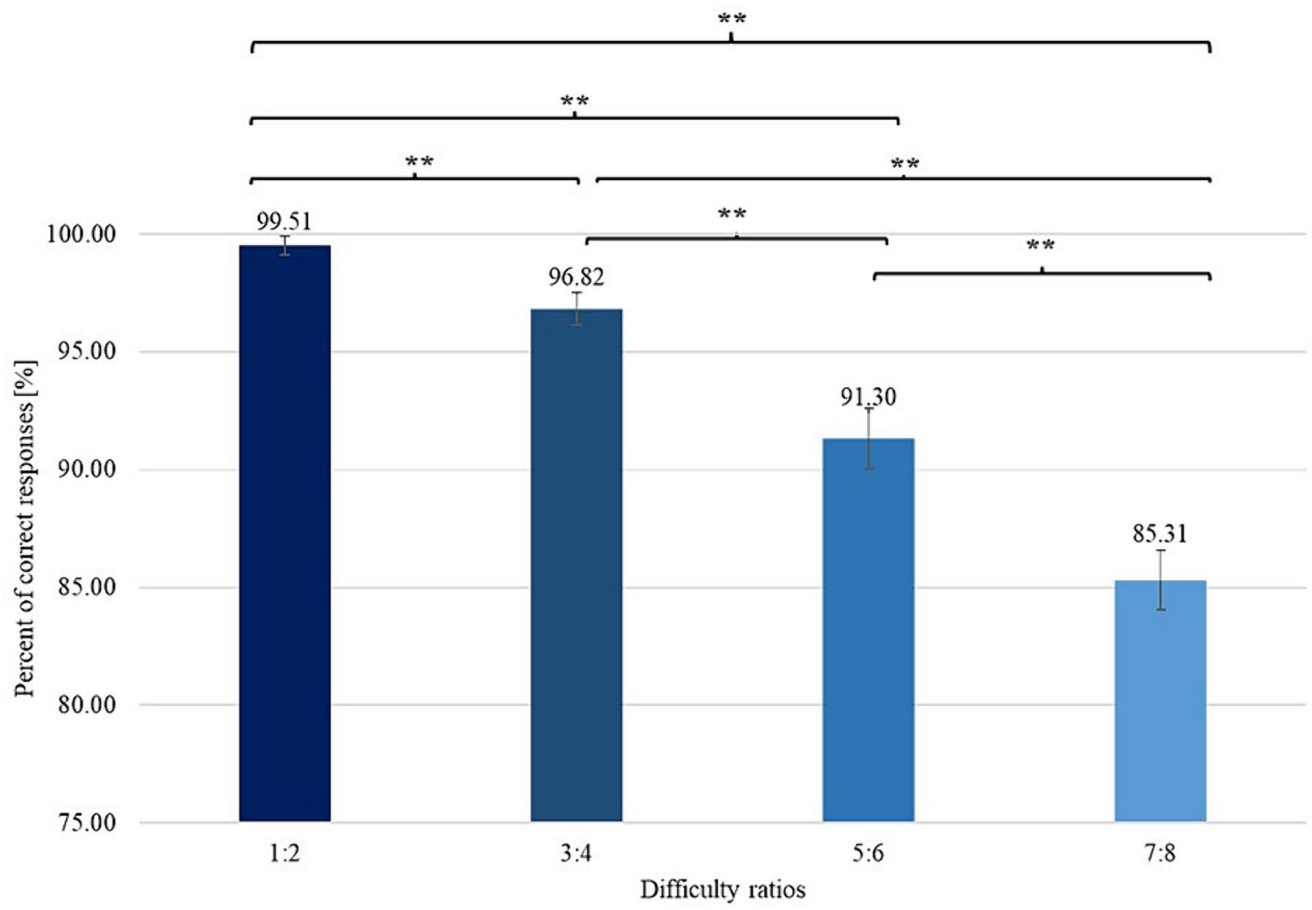
Differences among PCRs obtained with varying difficulty ratios with error bars (standard error of the mean, SEM). **signifies level of statistical significance (*p* is below 0.01).

**Figure 4:**
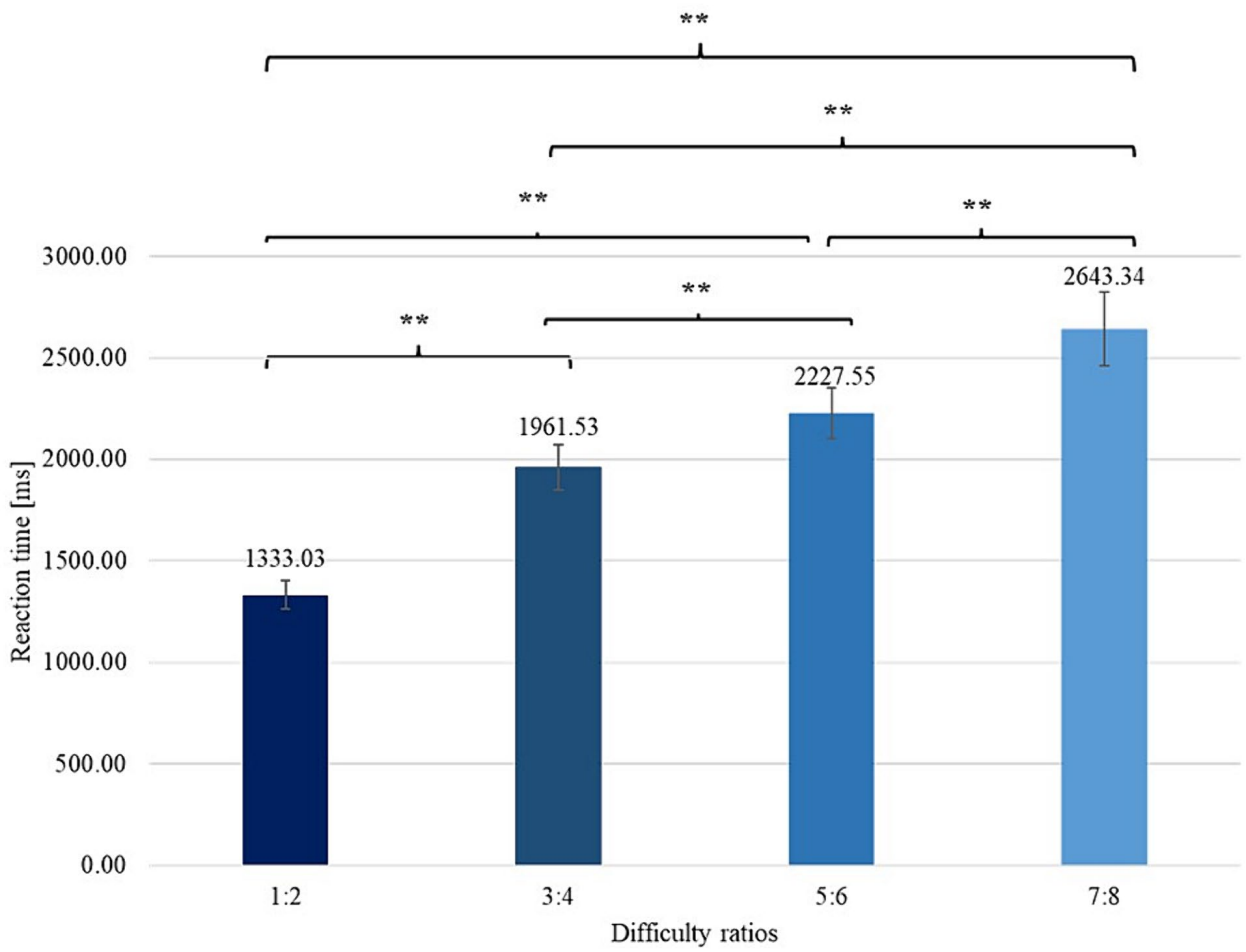
Differences between RTs obtained in particular difficulty ratios with error bars (SEM). **signifies level of statistical significance (*p* is below 0.01).

To verify the presence of differences between mean RT (and PCR) measured for different difficulty ratios, we conducted analyses of the variances in the averages between the means of RT and PCR as the dependent variables. These analyses showed the existence of significant differences in terms of the variables tested among all the difficulty conditions. The results showed that the simplest variant was the ratio of 1:2 and that the most difficult variant was the ratio of 7:8, as shown in the graphs (Figures 3, 4). Detailed results are included in Table 1.

**Table 1:** Mean values of RT and PCR for different ratio values of sets (varying difficulty of the task).

**Dependent variable**	**Numericities ratio differences**	**Statistic**	***p***	***d-Cohen***
PCR	1:2 and 3:4	*Z* = −4.030	<0.01	–
	1:2 and 5:6	*Z* = −5.360	<0.01	–
	1:2 and 7:8	*Z* = −5.804	<0.01	–
	3:4 and 5:6	*Z* = −4.971	<0.01	–
	3:4 and 7:8	*Z* = −5.786	<0.01	–
	5:6 and 7:8	*Z* = −4.459	<0.01	–
RT	1:2 and 3:4	*Z* = −5.905	<0.01	–
	1:2 and 5:6	*Z* = −5.905	<0.01	–
	1:2 and 7:8	*Z* = −5.905	<0.01	–
	3:4 and 5:6	*t* = −6.964	<0.01	−1.027
	3:4 and 7:8	*Z* = −5.818	<0.01	–
	5:6 and 7:8	*Z* = −4.834	<0.01	–

PCR, percent of correct responses; RT, response time [ms].

### Group differences analysis

To test the differences between participants with higher levels of overall cognitive functioning and those with lower levels of overall cognitive functioning, we further divided the participants into two groups in terms of their scores on the MoCA scale. The cut-off point for this scale, as proposed by Nasreddine et al. (2005), is 26 points. We chose this procedure because this scale is considered more difficult than the MMSE scale, where all participants included in the analyses scored at least 26 points. After taking into account the cut-off criterion, we defined the overall cognitive functioning of 29 people as decreased. In the case of another group (18 participants), their overall cognitive functioning was at the normal level for their age. The analyses verified whether the general level of cognitive functioning distinguishes the participants in terms of their PCR and RT while performing numerosity comparison task, as well as in terms of the cognitive strategies that they utilized. Analyses were separately conducted for the overall numerical task, as well as for particular difficulty variants (1:2, 3:4, 5:6, and 7:8). Because the results did not have a normal distribution, we decided to use the non-parametric Mann–Whitney test. The conducted analyses showed no significant differences between the distinguished groups in terms of RT and PCR. The only statistically significant differences were in the cognitive strategies applied by each group. An analysis of the overall results (all difficulty variants) show a significant difference among the groups regarding the frequency of use of a cognitive strategy based on two visual aspects of the presented sets - the distance between the dots and the total area of the set (*Z* = −2.392, *p* = 0.017). In the variant of difficulties with the ratio of dots in sets of 1:2, the differences were related to the most complex strategy, based on the size of the dots, the distance between them and the total surface area of the sets (*Z* = −2.044, *p* = 0.041). When the ratio of dots was 5:6, cognitively normal functioning participants were more likely to apply strategies using the size of the dots and the total area of the set (*Z* = −2.405, *p* = 0.016) and the group of strategies based on two different visual features of the sets of dots (*Z* = −1.957, *p* = 0.050). However, the analyses showed no statistically significant differences for tasks in which the ratios of dots were 3:4 and 7:8.

### Correlational analysis

To analyze the relationships among the areas under study (numerosity comparison ability, cognitive functioning, and cognitive strategies), we conducted correlation analyses for selected variables from the above-mentioned areas. The analyses showed no statistically significant correlations between age and education and between scores on cognitive tests and behavioral indicators of numerosity comparison ability. Importantly, for the aims of the reported study, the PCR and RT variables during the numerosity comparison task showed several significant correlations with cognitive scales performance and cognitive strategies. The strength of these relationships ranged from weak to moderate. For the first mentioned analyses, there were positive correlation between PCR and attention task in the MMSE and there were also negative correlation between PCR and short-term memory tasks in the MMSE. RT correlates negatively with visuospatial tasks in the MoCA and short-term memory in the MMSE. Detailed results are included in Table 2.

**Table 2:** Results of correlational analysis between numerosity comparison task and cognitive scales performance.

	Attention (MMSE)	Short-term memory (MMSE)	Visuospatial tasks (MoCA)
PCR	0.336*	−0.385**	–
RT	–	−0.324*	−0.302*

*p < 0.05; **p < 0.01.

There were also significant correlations between the MMSE and MoCA results and the frequency of using cognitive strategies – overall results in MoCA correlate positively with total surface area and distance between dots strategy. Performance on the short-term memory task correlates negatively with the use of the size of dots strategy and positively with total surface area strategy. Moreover, there were positive correlations between the long-term memory task on the MoCA scale and the total surface area and distance between the dots strategy and between the most complex cognitive strategy related to the size of dots, distance between them and total surface area. Detailed results are included in Table 3.

**Table 3:** Results of correlational analysis between frequency of using cognitive strategies and MoCA results.

	Overall results	Short-term memory task	Long-term memory task
Total surface area and distance between dots strategy	0.363*	–	0.421**
Size of dots strategy	–	−0.393**	–
Total surface area strategy	–	0.319*	–
Size of dots, distance between them and total surface area strategy	–	–	0.329*

*p < 0.05; **p < 0.01.

There was also a positive correlation between the results of the attention task in the MMSE and distance strategy and a negative correlation between the same task in MMSE and the total surface area strategy. We also found a negative correlation between the short-term memory task in the MMSE and the lack of cognitive strategy during the numerosity comparison task. Detailed results are included in Table 4.

**Table 4:** Results of correlational analysis between frequency of using cognitive strategies and MMSE results.

	Attention	Short-term memory
Distance strategy	0.290*	–
Total surface area strategy	−0.288*	–
Lack of cognitive strategy	–	−0.373

*p < 0.05; **p < 0.01.

A further part of the correlation analyses concerned the relationships between the cognitive strategies employed by the participants and the overall PCR and RT in the numerosity comparison ability task. There was positive correlation between PCR and lack of cognitive strategy and there was also negative correlation between PCR and the total surface area of dots strategy. The RT correlates positively with the lack of cognitive strategy and a size of dots strategy and negatively with total surface area of dots strategy. Detailed results are included in Table 5.

**Table 5:** Results of correlational analysis between overall PCR and RT in numerosity comparison task and cognitive strategies employed by the participants.

	Lack of cognitive strategy	Size of dots strategy	Total surface area of dots strategy
PCR	0.514***	–	−0.405**
RT	0.566***	0.385**	−0.374*

*p < 0.05; **p < 0.01; ***p < 0.001.

A series of correlation analyses were also conducted for PCR and RT in each variant of task difficulty and the level of cognitive task performance and cognitive strategies used by the participants – these significant results are reported in Supplementary Table S3. This part of the analyses reveals that RT across difficulty variants was most often associated positively with the lack of cognitive strategy and size of dots strategy and correlated negatively with total surface area strategy. PCR was most often correlated negatively with total surface area strategy and also with the lack of cognitive strategy but here the direction of correlation differs due to difficulty ratio variant. There were no significant relationships between PCR or RT and strategies related to distance, shape, size of dots and distance, size of dots and shape, size of dots and total surface area, distance and total surface area and size of dots, distance and total surface area. For the analyses of correlations between cognitive functioning and behavioral indicates from the numerosity comparison task, the results showed relationships between PCR and a task measuring attention on the MMSE scale (7:8 ratio variant) and short-term memory resources in the 3:4, 5:6, and 7:8 ratio variants. RT was correlated with performance on a task measuring visuospatial functioning in the MoCA scale (3:4 and 7:8 ratio variants), as well as with short-term memory resources in the 3:4, 5:6, and 7:8 ratio variants. There were no significant relationships between the overall cognitive test results and PCR and RT for different difficulty ratio variants.

### Regression analysis

For a better understanding of the relationship between indicators of numerosity comparison ability and cognitive functioning, we performed a series of stepwise regressions with RT and PCR scores as dependent variables and the results from cognitive functioning scales and type of cognitive strategies used by participants as predictors. We selected this regression method to explain numerosity comparison ability with as few variables as possible. We report models in which we found at least one significant predictor for scores in the whole task (we do not separately report results for each difficulty variants).

#### RT and cognitive functioning

In the analysis of the model with RT and cognitive functioning measures on the MMSE scale, the final model included only the effect of the short-term memory task [*R^2^* = 0.91; *F*(1, 46) = 4.485, *p* = 0.40]. This model explained 9% of the variance in the results. In the model with RT and cognitive functioning measures in the MoCA scale, an effect of visuospatial task was observed [*R^2^* = 0.93; *F*(1, 46) = 4.639, *p* = 0.037]. This model also explained 9% of the variance in the results.

#### PCR and cognitive functioning

In the analysis of the model with PCR and cognitive functioning measures in the MMSE scale, the final model included an effect of short-term memory and attention task [*R^2^* = 0.213; *F*(1, 46) = 5.958, *p* = 0.005]. A statistically significant model explained 21% of the variance in the results. The model of PCR and cognitive functioning measures in the MoCA scale was not found.

#### RT and cognitive strategies

In the analysis of the model with RT and cognitive strategies used by participants, the final model included the total surface area strategy [*R^2^* = 0.201; *F*(1, 46) = 11.351, *p* = 0.002]. A statistically significant model explained 20% of the variance in the results.

#### PCR and cognitive strategies

In the analysis of the model with PCR and cognitive strategies used by participants, the final model included the total surface area strategy [*R^2^* = 0.176; *F*(1, 46) = 9.579, *p* = 0.003]. A statistically significant model explained 17.6% of the variance in the results.

## Discussion

The results obtained in the study are important for the methodology of further ongoing projects and the future direction of research on numerical cognition and its relationship to the cognitive functioning of older people.

With regard to the first hypothesis that we formulated, we can state that it has been completely confirmed, which is evident in the results. Statistically significant differences among all difficulty variants are observed in the case of both RT and PCR. The direction of these differences coincides with the successive pairs of difficulty variants that are compared - the shortest RT and the highest PCR values are in the variant where the ratio of dots is 1:2, and the slowest RT with the lowest PCR are in the variant with a ratio of 7:8, where the differences between the numerosity of sets are 1 or 2 dots. These results show the validity of distinguishing these different levels of difficulty in research and during the conducted statistical analyses which may be attributed to the varying complexity of the cognitive task to be performed. The level of difficulty of the task, which is understood as the amount of information to be processed among the elderly people, may result in slower and less efficient processing of this information in reference to theories that indicate a general decline in cognitive processing with age (Salthouse, 1996; Craik and Salthouse, 2011). Further analyses of intergroup differences examined the variation in RT and PCR, as well as the cognitive strategies used in the two specified groups of participants (based on the MoCA scale cut-off). The results showed no significant differences between the groups in the case of RT and PCR, which may be attributable to the notion that the MoCA scale is mainly used to measure the general level of cognitive functioning, which, based on the results of another scale (MMSE), can be assessed as at least good for each of the test participants. Therefore, subtle differences in cognitive functioning may not have been apparent during the performance of the numerosity comparison task. The level of difficulty of this task may not have been challenging for the participants (the overall RT and PCR averages indicate a ceiling effect in the task). However, the results indicate significant differences between the groups in terms of the cognitive strategies used by the participants. Here, it is obvious that individuals with a higher overall level of cognitive functioning (minimum MoCA score of 26 points) are significantly more likely than those participants with lower levels of cognitive functioning to use cognitive strategies based on a minimum of two visual aspects of the sets of dots presented to them. Moreover, for the simplest difficulty variant (ratio of 1:2), the cognitive resources available to this group were reflected in the most complex cognitive strategy that they applied more often - consisting of all three visual aspects of the presented sets of elements (size of dots, total surface area of the set of dots and distances between dots). The significantly higher scores on the scale examining the overall level of cognitive functioning may reflect the ability to simultaneously process information from more sources of information (Glisky, 2007), which could be precisely reflected in the use of more complex cognitive strategies. Moreover, these differences may be explained by a higher level of consideration of one’s own behavior, which is associated with a higher level of cognitive functioning (Chen et al., 2005; Ready et al., 2006). Notably, during task performance, the participants paid precise attention to the difficulty of determining their own cognitive strategies themselves and not to the difficulty of the task itself. This finding further shows that this research method may be particular important for determining the state of cognitive impairment of the elderly people (Lemaire and Arnaud, 2008). Also in line with these results are the findings of an earlier study (Roquet and Lemaire, 2019), in which the ability of numerosity comparison was tested in two age groups – among adults (mean age = 21.1) and among older people (mean age = 74.8). The results of this study showed that older people were significantly more likely to choose simpler cognitive strategies (based on only one visual aspect of the presented stimuli) than younger adults - this difference was evident in the non-concurrent variant of the task, which requires participants to engage more of their available cognitive resources (Cappelletti et al., 2014; Clayton et al., 2015; Gilmore et al., 2016). Due to cognitive load during executing non-concurrent variant of the task, this result may provide indirect evidence of a link between the cognitive functioning of different age groups and the cognitive strategies they use. However, this is a hypothesis that needs to be verified in future studies involving different age groups.

The correlation analyses that we conducted among the variables allowed us to partially confirm our second hypothesis. The results of the analyses indicated no relationship between the overall level of cognitive functioning and the RT and PCR during the numerosity comparison task. The lack of significant relationships between the cognitive scales (MMSE and MoCA) and the behavioral indicators (RT and PCR) shows that these tools are insufficient to identify the cognitive functions underlying the correct execution of the numerosity comparison task. However, the revealed correlations show that memory as well as the visual–spatial and attention processing of the presented material can play a role in the speed and correctness in the completion of a numerosity comparison task, which allows us to partially confirm our hypothesis. The importance of these cognitive domains in this case is explained by the need to efficiently and quickly process the visual information presented to the participants during the task. Both attentional resources and visuospatial processing of this information are particularly important during the performance of any task in which complex visual information is presented (Carr and Bacharach, 1976; Coull et al., 1996; Carrasco and McElree, 2001). Moreover, greater memory resources make it possible to more effectively remember the information obtained when analyzing and processing individual sets of dots (here, the numerosity of each set). This efficiency is evident in faster responses (reanalysis of the counts of the sets is not necessary) but the results are inconclusive in term of correctness. The results obtained in the study contradict the potential assumption that greater available working memory resources would be associated with higher correctness in performing the task of numerosity comparison. Greater resources in this area of cognitive functioning should enable selection of the set that is actually larger. According to Baddeley and Hitch’s (1974) model, one of the elements of working memory - the visual–spatial sketchpad, which is responsible for storing visual information about the stimuli being processed - is particularly important for this purpose. The negative direction of this correlation in our study can be explained by methodological considerations of the project, more specifically, the cognitive functioning scales (MoCA and MMSE) used in the research, in which single tasks are often used to measure cognitive resources. Accordingly, this result indicates further directions for the studies, in which the character of the correlation between working memory and the numerosity comparison ability should be verified with use of more detailed tests of cognitive functioning. The importance of the indicated areas of cognitive functioning for the level of performance on individual numerical tasks has already been demonstrated in earlier studies, including memory (Imbo and Vandierendonck, 2007; Imbo and LeFevre, 2010; Cavdaroglu and Knops, 2016), spatial skills (Zhang and Lin, 2015; Cornu et al., 2017), attention (Dormal et al., 2014; Mathieu et al., 2016; Ben-Shachar and Berger, 2018) or selected executive functions (Cragg and Gilmore, 2014; Han et al., 2016; Nemati et al., 2017).

Another part of the correlation results concerned the relationships between the cognitive strategies used by the participants and the PCR and RT in the numerosity comparison ability task. Here, we also obtained some results that indicated that the choice of certain cognitive strategies was related to RT and PCR. We observed that the lack of cognitive strategy or the strategy connected with only one visual feature correlates to a longer RT but that a more complex strategy (related to two visual features) is positively correlated to RT. Therefore, we can assume that using a more complex cognitive strategy in the paradigm that we utilize will be associated with faster resolution of subsequent trials. However, we also obtained results that indicate that the lack of any cognitive strategy is related to a higher PCR and, simultaneously, that implementation of a strategy related to the total surface area of dots strategy is negatively correlated with the PCR. The results also conflict with our assumptions, which we can explain, among other things, by the necessity to separate the cognitive resources possessed by the participants into direct performance of the numerical task and the use of cognitive strategies. When these resources were reduced, it became necessary to transfer one’s abilities and resources to the performance of the task (perhaps with the simultaneous abandonment of the support of any cognitive strategy). On the other hand, there is also the possibility that the category ‘lack of cognitive strategy’ includes cases when, contrary to instructions, participants decided to count dots in individual sets, which must have impacted the increasing RT and PCR of task performance. The last part of the correlational analyses also showed that cognitive resources are related to selected cognitive strategies. In particular, the results showed that the overall level of cognitive functioning was reflected in the more frequent use of more complex strategies. Furthermore, specific aspects of cognitive function, including short-term memory strategies (size of dots and total surface area) and long-term memory strategies (total surface area, distance between dots, size of dots, distance between dots, and total surface area), as well as attentional resources (distance strategy and total surface area strategy), played a significant role in the utilization of these strategies. In addition, better short-term memory resources were associated with less frequent responses without the use of cognitive strategies. These results, similar to previous results, again indicate the special importance of memory and attentional resources in processing numerical information – here, in numerosity comparison. The previously mentioned cognitive resources probably enable more efficient processing of this information, which in our study was reflected in the cognitive strategies used by the participants. In summary, significant relationships between overall performance and the specified task in the MoCA and MMSE scales shows that better cognitive functioning allows the use of more complex cognitive strategies, which can ultimately lead to higher task performance.

To avoid limiting the performed statistical analyses to correlational analyses, which only report on the connections among variables, we also conducted a series of regression analyses. On the basis of these analyses, we were able to develop several models that explain the RT and PCR during the numerosity comparison task not only by the level of particular areas of cognitive functioning (short-term memory, attention and visual–spatial processing) but also by means of the cognitive strategies utilized by the participants, which we precisely treat here as a reflection of the level of cognitive resources (Pressley and Hilden, 2006). We observe the potential causes and sources of these phenomena in the same results of the earlier study that we cited in interpreting the correlational analyses - here, however, we are able to point to cause-effect relationships of the analyzed variables. With these results, we know that we have the ability to explain even specific and very basic numerical abilities with selected higher cognitive functions. Moreover, the obtained regression results show the possible utility of the described research paradigm in the diagnosis and prognosis of deficits in cognitive functioning. However, this area requires further research.

### Limitations of the study

According to the pilot nature of the study, there are several limitations that may have affected the results. The first limitation is the size of the research sample, which amounted to 50 participants (after excluding some participants, we used the data of 47 participants for the statistical analyses). Furthermore, as statistics related to the level of education of these people show, this was a specific group of elderly people who often maintain a high level of mental and social activity despite their age. It was easy to encourage the participation of this group of seniors in scientific projects. Therefore, our results may be a starting point for further scientific research, but it may be difficult to generalize them to the entire population of older people. The second limitation related to the pilot nature of the research was the choice of methods to measure the level of cognitive functioning of the participants. The MMSE and MoCA scales allow the study of cognitive functioning at a general level, while specific cognitive functions are measured with short and single tasks. Therefore, to study investigate possible cognitive deficits in future projects, it is necessary to apply more specified methods with verified psychometric properties (e.g., Color Trails Test, Benton Visual Retention Test). The third limitation is related to the results obtained in the numerical task used to measure numerosity comparison ability. As the findings show, the initial level of difficulty used in the study was unsuitable for the cognitive abilities of the participants, which resulted in their obtaining very high results (visible both in the RT of responses and the PCR). The last limitation that may have affected the results of the research project was the difficulty encountered among the participants in formulating answers related to the cognitive strategies that they used during the numerosity comparison task. Despite the preparation of detailed instructions, some of the participants were unable to formulate answers that fit the developed answer key (e.g., intuition, first impression, and counting dots). For these individuals, these responses were judged to be the lack of cognitive strategy. This procedure was chosen to avoid giving instructions that overtly indicated the answers, which could significantly misrepresent the results.

### Directions of further research

As there are still many uncertainties about the level of numerical skills and their dependence on the level of cognitive functioning, further scientific research in this field is needed. One research are that could provide significant results is the implementation of technologies into cognitive strategy research that enable the collection of data that are more objective than the verbal responses of participants (e.g., eye-tracking). Furthermore, there is a need for studies that involve varied clinical groups (e.g., mild cognitive impairment, Alzheimer’s disease), which will allow us to compare these groups and observe possible changes in numerical abilities over the progression of neurodegenerative diseases, as well as to conduct similar research by including younger participants, which will allow to directly examine the influence of age on the studied ability and the cognitive strategies used during the examination. Longitudinal projects, which require substantial work and motivation of the participants, are also becoming important in the same area. The data collected in this research should provide answers to questions about the prognostic, diagnostic and therapeutic values of individual numerical skills in the process of cognitive aging.

## Data availability statement

The raw data supporting the conclusions of this article will be made available by the authors, without undue reservation.

## Ethics statement

The studies involving humans were approved by Local Ethics Committee of the Faculty of Philosophy and Social Sciences on Nicolaus Copernicus University in Torun. The studies were conducted in accordance with the local legislation and institutional requirements. The participants provided their written informed consent to participate in this study.

## Author contributions

JS: Writing – review & editing, Writing – original draft, Investigation, Conceptualization, Formal analysis. MG: Writing – review & editing, Conceptualization. JM: Writing – review & editing, Software. AT: Writing – review & editing, Conceptualization.
