# Supplementary material for: Numerosity comparison, cognitive strategies, and general cognitive functioning in older people
Source: Front Psychol. 2024 Apr 2;15:1340146. doi: 10.3389/fpsyg.2024.1340146 (PMC11020078; doi:10.3389/fpsyg.2024.1340146)
Supplement: Supplementary file 1 [file Data_Sheet_1.docx]

# Supplementary Material

**Instruction given to participants before the numerosity estimation task.**

The task you will be performing consists of a number of trials, which have been divided into several parts to allow you a short break. Each task should be performed as quickly as possible, but at the same time as correctly as possible.

During the task you will see two sets of dots on the computer screen. Your task is to choose, as quickly as possible, the set which, in your opinion, is more numerous - there are more dots in it. At the same time, please remember not to count the presented sets of dots, but to estimate their number. To select the left set you need to press the Z key, and the right set - the M key. After each such selection I will ask you what visual characteristics of the presented sets of dots influenced your choice of the larger set of dots. For example, these could be the size of the dots, their surface area, the distances between them or their shape. There may be more of these features than I have just listed. I also ask that between tasks you return with your eyes to the figure that will appear in the center of the screen (the white rhombus). The first part will be a training for you so that you have the opportunity to practice the task. Do you perhaps have any questions or concerns? Are you ready?

The next parts will be the proper part of the test. Please try to choose as quickly and correctly as possible those sets where there will be a greater number of dots.

Question asked to explore cognitive strategy – What visual features of the sets presented on the computer screen influenced your choice of a larger set of dots?

Table 1. Descriptive statistics of variables reported in the study.

| **Variable** | ***n*** | ***Mean*** | ***SD*** | ***Min*** | ***Max*** |
| --- | --- | --- | --- | --- | --- |
| MoCA overall result | 47 | 24.83 | 2.23 | 21 | 29 |
| Visuospatial tasks (MoCA) | 47 | 3.68 | 1.02 | 2 | 5 |
| Long-term memory (MoCA) | 47 | 2.62 | 1.33 | 0 | 5 |
| MMSE overall result | 47 | 28.36 | 1.31 | 26 | 30 |
| Attention (MMSE) | 47 | 4.47 | 0.95 | 2 | 5 |
| Short-term memory (MMSE) | 47 | 2.11 | 0.91 | 0 | 3 |
| PCR overall | 47 | 93.13 | 4.98 | 80 | 100 |
| PCR 1:2 ratio variant | 47 | 99.51 | 2.65 | 82.05 | 100 |
| PCR 3:4 ratio variant | 47 | 96.82 | 4.62 | 80.49 | 100 |
| PCR 5:6 ratio variant | 47 | 91.30 | 8.70 | 63.16 | 100 |
| PCR 7:8 ratio variant | 47 | 85.31 | 8.60 | 68.29 | 100 |
| RT overall [ms] | 47 | 2027.78 | 789.97 | 802.35 | 4291.68 |
| RT 1:2 ratio variant [ms] | 47 | 1333.03 | 474.51 | 687.24 | 3240.11 |
| RT 3:4 ratio variant[ms] | 47 | 1961.53 | 759.35 | 759.90 | 4203.12 |
| RT 5:6 ratio variant[ms] | 47 | 2227.55 | 831.44 | 879.46 | 4568.17 |
| RT 7:8 ratio variant[ms] | 47 | 2643.34 | 1221.50 | 885.87 | 5800.33 |

Table 2. Frequency of use of particular cognitive strategies by participants in the study

| **Cognitive strategy** | ***n*** | ***Mean*** | ***SD*** | ***Min*** | ***Max*** |
| --- | --- | --- | --- | --- | --- |
| Lack of strategy | 47 | 17.66 | 23.39 | 0 | 100 |
| Distance strategy | 47 | 10.67 | 19.78 | 0 | 83.1 |
| Size of dots strategy | 47 | 39.88 | 28.78 | 0 | 100 |
| Total surface area strategy | 47 | 17.70 | 23.25 | 0 | 93.8 |
| Shape strategy | 47 | 2.11 | 11.15 | 0 | 76.3 |
| Size of dots and distance strategy | 47 | 2.59 | 4.51 | 0 | 26.3 |
| Size of dots and shape strategy | 47 | 0.199 | 0.74 | 0 | 4.4 |
| Size of dots and total surface area strategy | 47 | 6.09 | 8.35 | 0 | 34.4 |
| Distance and total surface area strategy | 47 | 1.81 | 3.77 | 0 | 18.8 |
| Size of dots, distance and total surface area strategy | 47 | 0.76 | 2.70 | 0 | 16.9 |

Table 3. Correlations between PCR or RT in each variant of task difficulty and other variables.

|  | RT (1:2) | PCR (1:2) | RT (3:4) | PCR (3:4) | RT (5:6) | PCR (5:6) | RT (7:8) | PCR (7:8) |
| --- | --- | --- | --- | --- | --- | --- | --- | --- |
| Visuospatial tasks (MoCA) | - | - | -0.306* | - | - | - | -0.294* | - |
| Attention (MMSE) | - | - | - | - | - | - | - | 0.409** |
| Short-term memory (MMSE) | - | - | -0.336* | - | -0.313* | - | -0.378** | - |
| Lack of strategy | 0.303* | -0.311* | 0.457** | - | 0.571** | 0.520** | 0.554** | 0.442** |
| Distance strategy | - | - | - | - | - | - | - | - |
| Size of dots strategy | 0.391** | - | 0.398** | - | - | - | 0.374* | - |
| Total surface area strategy | -0.333** | - | -0.308* | - | -0.357* | -0.369* | -0.434** | -0.341* |

* - *p* < 0.05; ** - *p* < 0.01
